# Stereology and three-dimensional reconstructions to analyze the pulmonary vasculature

**DOI:** 10.1007/s00418-021-02013-9

**Published:** 2021-07-16

**Authors:** Christian Mühlfeld

**Affiliations:** 1 Institute of Functional and Applied Anatomy, Hannover Medical School, Carl-Neuberg-Str. 1, 30625 Hannover, Germany; 2 Biomedical Research in Endstage and Obstructive Lung Disease Hannover (BREATH), Member of the German Center for Lung Research (DZL), Hannover, Germany; 3 Research Core Unit Electron Microscopy, Hannover Medical School, 30625 Hannover, Germany

**Keywords:** Pulmonary vasculature, Alveolar capillary network, Design-based stereology, Electron microscopy, 3D reconstruction

## Abstract

The pulmonary vasculature consists of a large arterial and venous tree with a vast alveolar capillary network (ACN) in between. Both conducting blood vessels and the gas-exchanging capillaries are part of important human lung diseases, including bronchopulmonary dysplasia, pulmonary hypertension and chronic obstructive pulmonary disease. Morphological tools to investigate the different parts of the pulmonary vasculature quantitatively and in three dimensions are crucial for a better understanding of the contribution of the blood vessels to the pathophysiology and effects of lung diseases. In recent years, new stereological methods and imaging techniques have expanded the analytical tool box and therefore the conclusive power of morphological analyses of the pulmonary vasculature. Three of these developments are presented and discussed in this review article, namely (1) stereological quantification of the number of capillary loops, (2) serial block-face scanning electron microscopy of the ACN and (3) labeling of branching generations in light microscopic sections based on arterial tree segmentations of micro-computed tomography data sets of whole lungs. The implementation of these approaches in research work requires expertise in lung preparation, multimodal imaging at different scales, an advanced IT infrastructure and expertise in image analysis. However, they are expected to provide important data that cannot be obtained by previously existing methodology.

## Introduction

**Fig. 1:**
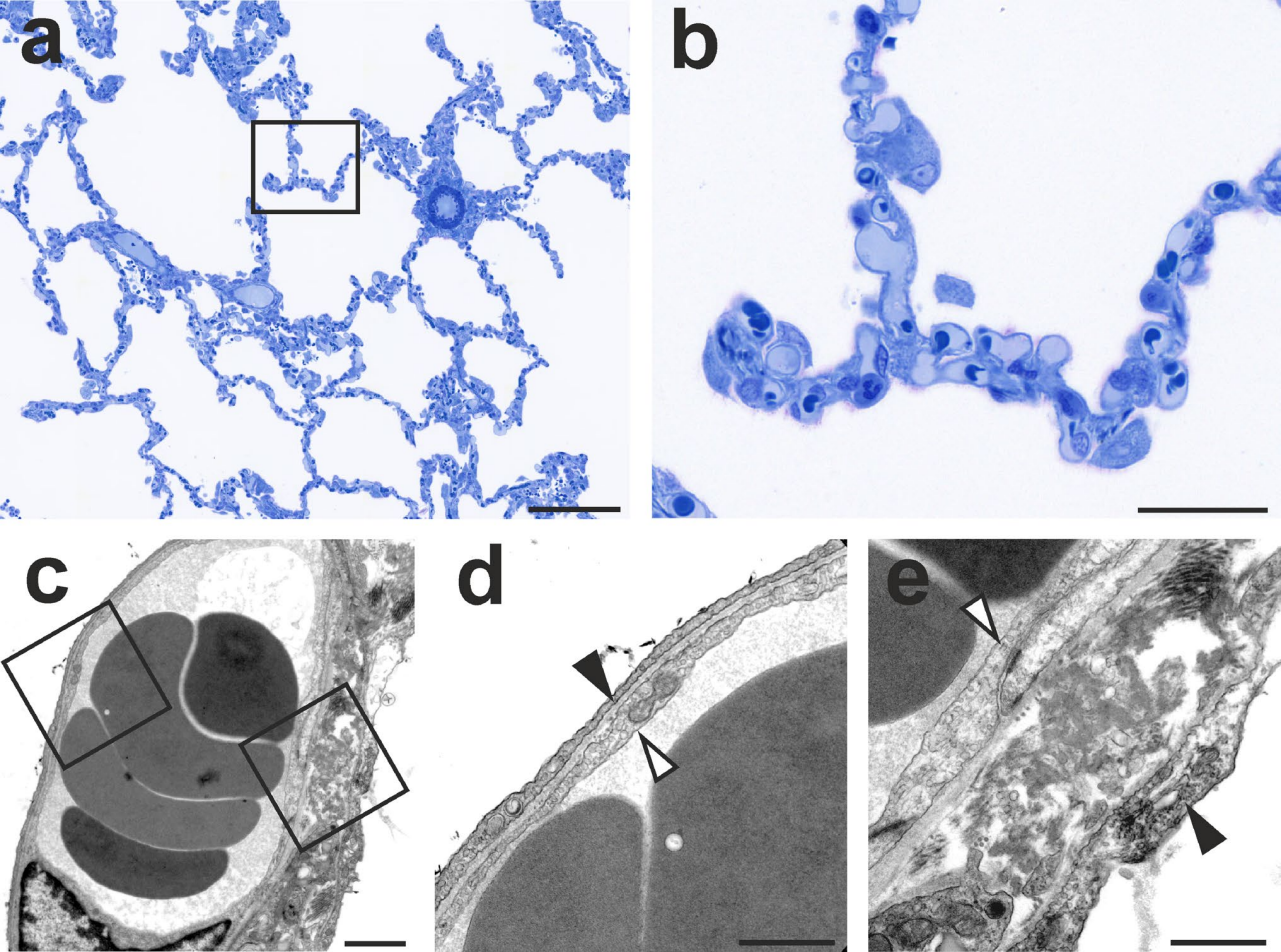
Alveolar capillaries in classical light and electron microscopy. **a** Low-power light micrograph of the gas-exchange region of a human lung sample embedded in glycol methacrylate, toluidine blue staining, 2 µm thick section. **b** Higher magnification of the box in **a** showing the dense network of capillaries in the interalveolar septa. **c** Transmission electron micrograph of an interalveolar septum of a human lung sample embedded in epoxy resin. **d** Higher magnification of the thin part of the air–blood barrier (left box in **c**). **e** Higher magnification of the thick part of the air–blood barrier (right box in **d**). The archived human lung sample was kindly provided by Prof. Ewald Weibel. Black arrows: alveolar epithelium; white arrows: capillary endothelium. Scale bars: **a** 200 µm; **b** 50 µm; **c** 2 µm; **d** and **e**, 1 µm

The gas-exchange function of the mammalian lung is closely linked to its structural composition (Weibel 2017). At the parenchymal level, the close structure–function relationship is recognizable in the large epithelial and endothelial surface area for gas exchange as well as the extremely thin barrier that separates air and blood (Gehr et al. 1978; Maina and West 2005). Therefore, conditions decreasing the surface area (e.g. emphysema) or increasing the barrier thickness (e.g. edema, fibrosis) have a direct adverse effect on gas exchange. Epithelial and endothelial surface are constantly and simultaneously in contact with air and blood, respectively, which makes the lung susceptible to pathogenic stimuli coming in through air and blood. The capillaries of the alveolar septa form a vast network of short, wide capillary segments that functions as a sheet of blood, only interrupted by pillars containing connective tissue (Fung and Sobin 1969; Sobin et al. 1970; Weibel 2009) (Fig. 1).

The blood reaches the lungs via the pulmonary arteries more or less in parallel with the airways. The veins draining the blood from the capillary bed run within interlobular septa and do not follow the arterial and airway paths. Like the airways and like arteries of the systemic circulation, the wall composition of the pulmonary arterial branches changes along the vascular tree. The pulmonary arteries are involved in important physiological processes such as hypoxic vasoconstriction (Von Euler and Liljestrand 1946; Grimmer and Kuebler 2017) as well as human diseases including bronchopulmonary dysplasia (Coalson 2003; Thébaud et al. 2019) and pulmonary hypertension (Farkas and Kolb 2013; Tuder 2017).

Thus, both the “conducting” blood vessels and the gas-exchanging alveolar capillary network (ACN) are essential for the physiological function of the lung and are critically involved in human diseases. Due to the close link between structure and function of the lung, (quantitative) morphological methods to analyze the different vascular compartments of the lung contribute to understanding the healthy and diseased lung (Weibel 2017; Ochs 2010; Ochs et al. 2016). Despite the long tradition of lung morphology and morphometry, several new developments have expanded the analytical spectrum of the pulmonary vasculature in recent years. They are the subject of this review article.

## Stereology

Design-based stereology is the gold standard of morphometric studies on the lung (Hsia et al. 2010)—it is, by the way, the gold standard of microscopic morphometry in general, but its application is most frequent in neuroscience, lung and kidney research (Nyengaard 1999; Puelles et al. 2014; Hsia et al. 2010; Schmitz and Hof 2005). Numerous detailed reviews on stereology of the lung are available (Bolender et al. 1993; Ochs 2006; Mühlfeld and Ochs 2013; Ochs and Mühlfeld 2013; Brandenberger et al. 2015; Knudsen et al. 2021), and their content will not be repeated here. In short, design-based stereology provides methods that help to obtain (theoretically) unbiased quantitative data about biological structures in the three-dimensional space from two-dimensional sections through the organ of interest. First-order stereological parameters include volume, surface area, length and number of structures, which are estimated as ratios (“densities”) in a small volume of the whole organ and then extrapolated to the reference volume. Thus, the estimates are finally given in total volume, surface area, length and number. Local stereological tools are also available for estimating volume- or number-weighted mean volumes of cells or organelles as well as mean thickness of barriers (Rasmusson et al. 2013; Gundersen 1988; Cruz-Orive 1987). In general, design-based stereology has a strong mathematical background which is based on stochastic geometry. One of the key principles of stereology is sampling (at various levels) to make sure that the results are representative of the whole reference volume (Mayhew 2008; Gundersen and Jensen 1987; Tschanz et al. 2014).

My own first study addressing the pulmonary vasculature stereologically is the story of a failure; however, I learnt so much during this process that I think it is worthwhile to tell it here. At that time, I had started working at Wolfgang Kummer’s lab at the Anatomy and Cell Biology Department of Gießen University, who had asked me to develop a stereological way of quantifying the innervation of organs such as the heart. The resulting methodological paper combined the estimation of the total length of immunohistochemically labeled nerve fibers at the light microscopic level and the mean number of axons per nerve fiber at the electron microscopic level (Mühlfeld et al. 2010a,^2011^). When archived material is used, the method can also be applied solely at the electron microscopic level, although less efficiently (Schipke et al. 2014). The method was also applied to the mouse trachea using a pre-embedding immunohistochemical approach (Graulich et al. 2014). Thrilled by their efficiency, I had planned to apply stereological length estimations to the alveolar capillaries in a set of archived lung samples of 14 species, including humans. The material which had been kindly provided by Ewald Weibel had earlier served for a comparative stereological study on alveolar epithelial type 2 cells and their surfactant-storing lamellar bodies (Wirkes et al. 2010). Looking at the literature, length estimation of blood vessels seemed to be a common tool in other organs such as heart, kidney and central nervous system (Mall et al. 1987; Bertram 1995; Dockery and Fraher 2007). I also found a few stereological studies that had applied length estimation to the alveolar capillaries of mammalian lungs (Knust et al. 2009; Wiebe and Laursen 1998; Wiebe et al. 2006; Howell et al. 2003, 2009). With the definition of a capillary profile as a lumen entirely surrounded by capillary endothelium, the estimation required counting of capillary profiles within the area of an unbiased counting frame (Gundersen 1977). The resulting data yielded a beautiful allometric relationship that fit well with other allometric data on the lung (Gehr et al. 1981). The results were supposed to be published in the Anatomical Record, which I owed a manuscript with the submission deadline quickly approaching. After having written the manuscript, I sent it to Ewald Weibel, whom I had listed as a co-author, and asked him for his opinion. His quick response basically said: “This is all nonsense. You can’t estimate length like this.” With the literature at my back I tried to argue against him, also because it remained unclear to me why it should not be possible, and I involved other well-renowned stereologists including Jens Nyengaard, Ute Hahn and Matthias Ochs. The emails that were sent around among the group of interested people exceeded by far the volume of the article that was finally written, and included many sketches to illustrate each one’s position. Finally, we agreed that it was indeed not possible to unbiasedly quantify the length of pulmonary capillaries, and demonstrated this on a set of five human lungs (Mühlfeld et al. 2010b). The reason for this is that the alveolar capillaries form a three-dimensional network (ACN) that does not meet the prerequisites for stereological length estimation. In a 3D network that is built to maximize the surface area by functioning as a sheet of blood, the component elements are nearly as long as wide, making an unbiased length estimation of the axial skeleton impossible. Thus, length estimations cannot be done unbiasedly because length is simply not a suitable characteristic to describe the network. To put it more bluntly: it only makes sense to estimate a parameter that is a natural 3D feature of the structure of interest. Although this was the main message of the manuscript, it is often cited for the biased estimates of capillary length given in it.

The abstract of the article closes with the sentence: “Until new methods are being developed, the unbiased estimates of capillary volume, and surface area should be preferred.” Among the basic global characteristics, the number of capillaries had not been investigated so far. The number of structures is usually not as closely linked to function as volume and surface area; however, it is often the basis for the definition of a disease state. For example, in emphysema the destruction of alveoli or in bronchopulmonary dysplasia, the disrupted alveolarization can only be accurately expressed by estimation of the alveolar number (Ochs et al. 2004; Hyde et al. 2004; Ochs 2014; Nardiello et al. 2017b). Furthermore, numerical changes often help to better understand the mechanisms behind a functional alteration of an organ. Therefore, it seemed that estimation of capillary number could be a suitable parameter. Here, it may be relevant to emphasize that the term number is frequently misused in the scientific literature by interpreting cells or vessels per area or microscopic field as a measure of number. Such data are prone to misinterpretation mainly for two reasons: (1) Because of the dimensional loss in sectioned samples, the number of particles is not represented within a single thin section. By the same token, the probability of a particle to be present in a 2D section depends on its extension perpendicular to the cutting plane, i.e. larger particles have a higher chance of being sectioned (for an example, see Mühlfeld and Ochs 2014). (2) The number of particle profiles per area is a relative parameter that depends not only on the number of particles but also on the reference space to which they are related. For example, in hypertrophic myocardium the number of cell profiles per microscopic field will certainly be smaller, but that does not necessarily mean that the total number of cells is smaller than in control myocardium.

In 1984, a famous Danish stereologist solved the question of number estimation under the nom de plume D.C. Sterio. When the letters of this name are rearranged, they form the name of the method proposed in this paper: the disector (Sterio 1984), which comes in two flavors, a physical and an optical one. Basically, the disector generates a counting volume from two physical sections or two focal planes within a thick section. The volume that lies between the two “sections” is the reference in which particles are counted based on a unique feature, namely their top or their bottom. If the top of a particle lies within the volume between the two sections, then this will be represented by the presence of the particle profile in the lower but not in the upper section. This method was later applied to more complex structures such as bone trabeculae (Gundersen et al. 1993), mitochondria (Kroustrup and Gundersen 2001; Eisele et al. 2008), alveoli (Ochs et al. 2004; Hyde et al. 2004) and renal glomerular capillaries (Nyengaard and Marcussen 1993). All of these complex estimators rely on the estimation of a measure of their connectivity by the Euler-Poincaré characteristic. Using this methodology, the following topological events are possible: (1) A particle profile is present in one but not in the other section of a disector (island). (2) Two separate particle profiles in one section are connected in the other one (bridge). (3) A ring-like particle profile is present in one section but closed in the other section (hole).

**Fig. 2:**
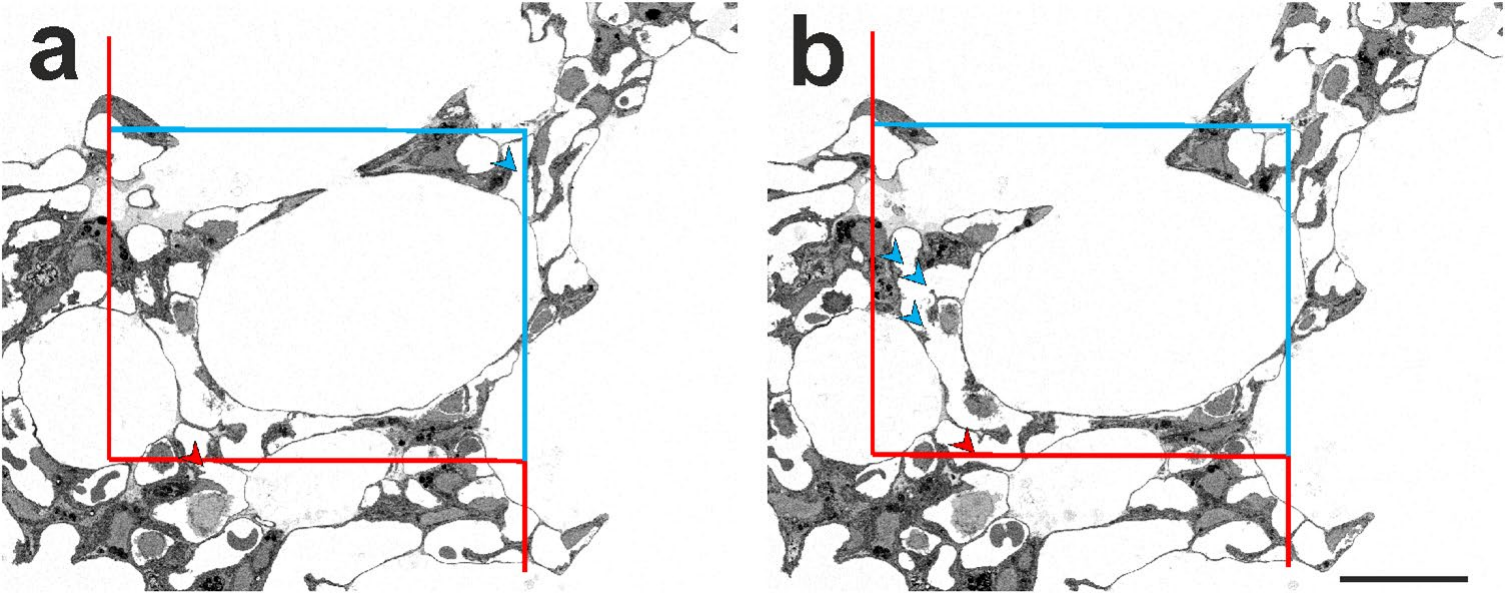
Physical disector images obtained by serial block-face scanning electron microscopy (SBF-SEM). **a**, **b** Two images of a larger data set obtained by SBF-SEM which was performed on a mouse lung sample embedded in epoxy resin (Durcupan™ ACM) according to the rOTO protocol (rOTO: reduced osmium tetroxide “thiocarbohydrazide” osmium tetroxide). The sections are 960 nm apart. An unbiased counting frame is projected onto the images with an exclusion line (red) and an inclusion line (light blue). Counting events (“bridges,” connections between capillaries in one of the images that are separated in the other image) are marked by light blue arrows. Bridges that touch the exclusion line are not counted (red arrows). Scale bar: 20 µm

Based on this, a workflow was established to estimate the Euler–Poincaré characteristic of alveolar capillaries on lung tissue of early postnatal and adult rat lungs (Willführ et al. 2015). A prerequisite is that the capillaries are widely opened. This can be achieved by fixation of the lungs by vascular perfusion (Gil and Weibel 1971) or by keeping the vascular pressure high during instillation fixation to prevent the collapse of the alveolar capillaries (Bur et al. 1985). The latter can be achieved by ligation of the incoming and outgoing blood vessels of the lung. After estimation of the lung volume by the Cavalieri estimator (Gundersen and Jensen 1987) or by fluid displacement/Archimedes’ principle (Scherle 1970), the lung is then sampled according to one of the established procedures such as systematic uniform random sampling (Gundersen and Jensen 1987), the fractionator (Gundersen 1986) or the smooth fractionator (Gundersen 2002). The samples need to be embedded in a way that prevents tissue shrinkage/deformation (Schneider and Ochs 2014) and allows the generation of very thin sections (1 µm or less). From the embedded tissue blocks, disector pairs can be generated which are then used to estimate the Euler number (Euler–Poincaré characteristic) of the capillary network. From this, the number of capillary loops within the network is estimated, a parameter which is equivalent to the number of tissue pillars in the sheet-like network of the ACN (Willführ et al. 2015) (Fig. 2). Although this makes the interpretation more difficult than the number of capillary segments between branching points, it provides very useful information to evaluate how changes in surface area or volume of capillaries are reflected in the 3D architecture of the ACN. The method awaits its first application to a biological question, and current projects of our work group are devoted to this. In a recent work on the pathogenesis of BPD it was shown that the decline of endothelial surface area precedes the decline of epithelial surface area in the hyperoxia model of BPD lending support to the vascular hypothesis that the stop of microvascular maturation causes the disorder in the alveolarization process (Appuhn et al. 2021). Further work on the Euler–Poincare characteristic of both alveoli and capillaries will provide further information on the relationship between microvascular development and alveolarization during lung development.

## 3D “reconstruction” and visualization of the alveolar capillary network

**Fig. 3:**
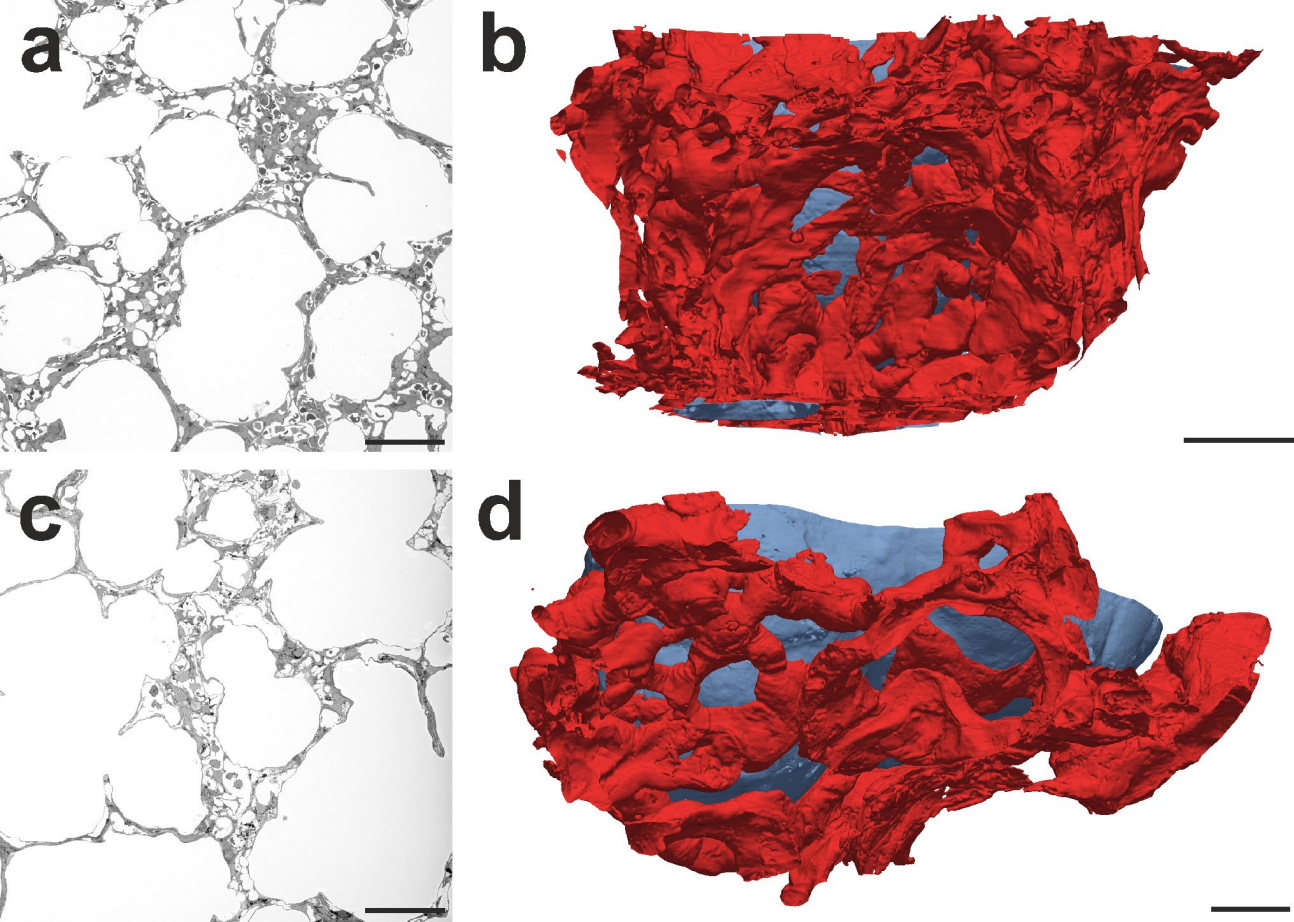
3D reconstruction of the alveolar capillary network from a serial block-face scanning electron microscopic data set. **a**, **c** 2D examples of the data set obtained by SBF-SEM which was performed on mouse lung samples embedded in Durcupan according to the rOTO protocol. **b**, **d** Segmented alveolar capillary network from two alveoli shown in the 2D images. The material was taken from a study comparing the capillaries of mice subjected to normoxia (**a**, **b**) or hyperoxia (**c**, **d**) during the first 14 postnatal days (Appuhn et al. 2021). Scale bars: **a**, **b** 50 µm; **c**, **d** 20 µm

One of the reviewers of the Willführ et al. (2015) paper had asked for a confirmation of the stereological method in a model where the number of capillary loops is known before, so that the stereological method could be verified. To address this point, the group of authors decided to perform a digital 3D reconstruction of the ACN in a sample of the rat lung. For this purpose, a sample embedded in epoxy resin was sectioned exhaustively into 1 µm thick sections and the sections were digitalized using a slide scanner. Roman Grothausmann, a physicist working at our institute at that time, already had extensive experience with such work and led this process which proved to be far more complicated than initially thought. One of the problems with 3D reconstructions arises from section artefacts (compression, stretching, folds etc.) or the loss of sections within the series which complicates the alignment of the data before the automated segmentation begins. Subsequent work using data generated by serial block-face scanning electron microscopy proved to be much easier in this respect as the data are not based on sections but on scans of the block surface (Fig. 3).

For an automated segmentation approach of the capillary network, the lungs need to be prepared in a similar way as for the Euler number estimation; however, in this case an optimal perfusion fixation is mandatory. Collapsed capillary segments or erythrocytes within the capillaries currently cannot be automatically identified by the algorithms, and lead to interruptions in the segmented network. Although erythrocytes can be manually removed from the segmentation, it is impossible to identify and reopen collapsed capillaries. Hence, it is useful to put extra effort into generating ideal perfusion fixation results before starting the segmentation procedure. The segmentation method which was subsequently described by Grothausmann et al. (2017) is based on a 3D watershed algorithm that distinguishes the segments “airspace lumen,” “blood vessel lumen” and “tissue barrier between air and blood.” Obviously, for a distinction between air and blood segments it is essential that the barrier between them is sufficiently preserved and resolved in the images. This is particularly difficult for the thin parts of the air–blood barrier where epithelium and endothelium are only separated by the fused basal lamina of both cell types which has a thickness of only a few hundred nanometers (Low 1953; Weibel 1971; Gehr et al. 1978). Artificial or true interruptions of the barrier affect the efficiency of the segmentation significantly. However, manual segmentation, for example by contour drawing (Kremer et al. 1996; Schneider et al. 2019), is not an alternative for larger parts of the ACN, as it is extremely time-consuming for this complex network. As mentioned above, the automated approach was originally performed on a series of 1 µm thick sections at the light microscopic level (Grothausmann et al. 2017). In the next step, the method was further applied to a data stack generated by serial block-face scanning electron microscopy of a neonatal mouse lung (Buchacker et al. 2019). During development the lung undergoes several stages: embryonic, pseudoglandular, canalicular, saccular and alveolar. In the saccular stage the primitive airspaces contain two layers of capillaries within the septa which are considered to be essential for the alveolarization process (Burri 1974; Schittny 2017). Rodents are physiologically born in the saccular stage of lung development, with alveolarization taking place after birth. In humans and other mammals, the alveolarization starts already before birth (Thurlbeck 1975). This makes mice and rats interesting models to investigate aspects of lung development that are part of intrauterine development in other species. However, it should be kept in mind that the lungs of term born rodents, despite their structural immaturity, are fully equipped to meet the functional demands of extrauterine life (Nardiello et al. 2017a). The segmentation of the SBF-SEM data showed that the term “double-layered capillary network” does not correctly describe the 3D characteristics of the capillary network in newborn mice. The dense network does not consist of two separate layers, which is often suggested in schematic drawings, but forms a 3D network with multiple connections between the two sides (“layers”) adjacent to the epithelial surface. The 3D model also allowed the visualization of the perfusion unit of an arteriole flowing out into the capillary network. Thus, it could be shown that the capillaries of many alveoli are connected and can be reached from various arterioles. Furthermore, intussusception as a mode of angiogenesis (Burri and Tarek 1990) can be visualized very well, in particular when virtual endoscopy or virtual reality are used for analysis. In the hyperoxia model of BPD in the mouse, Appuhn et al. (2021) used this methodology to describe the dysmorphic capillary network in BPD more closely.

## Combining stereology and 3D reconstruction of the arterial vessel tree

Although some challenges in analyzing the alveolar capillaries have been mentioned above, one thing is easy: identifying them. This does not necessarily hold true for the arterial or venous tree. Once a lung has been sectioned and is looked at under the microscope, several blood vessel profiles are visible. At best it is possible to distinguish between arteries and veins, although in individual cases this can also be difficult, in particular for the smaller branches. Also, based on the wall thickness, the diameter and the localization closer to the hilus or within the parenchyma, it can be guessed that a certain profile belongs to the more proximal or peripheral arteries. However, there is a large spectrum of arterial branches in between for which it is impossible to define its position within the arterial tree from a single two-dimensional section. Therefore, morphometric studies are often either very unspecific (all arterial/venous branches are compiled in a single compartment) or rely on a group of vessels that seem to be easy to identify. Within a homogeneous cohort (e.g. lab animal control group) the grouping of arteries according to their diameter or wall thickness may lead to consistent results. However, when these data are used to draw a comparison with an experimental group with altered vascular characteristics, it may well be that different types of arteries are compared with one another. The difficulty in defining a coherent vascular compartment can also lead to biased observations (van Suylen et al. 1998). In studies on pulmonary hypertension, a frequently used readout parameter is the “number” of non-muscularized, partially muscularized or fully muscularized peripheral pulmonary arteries (Klinger et al. 1999; Weissmann et al. 2007). Although this procedure solves the practical problem of putting different types of arterial branches into one compartment, it may lead to the conceptual bias that pulmonary hypertension is only a disease of the intra-acinar arteries, although other arterial generations are known to be affected as well (Ohara et al. 1991; Wang et al. 1991; Tajsic and Morrell 2011).

**Fig. 4:**
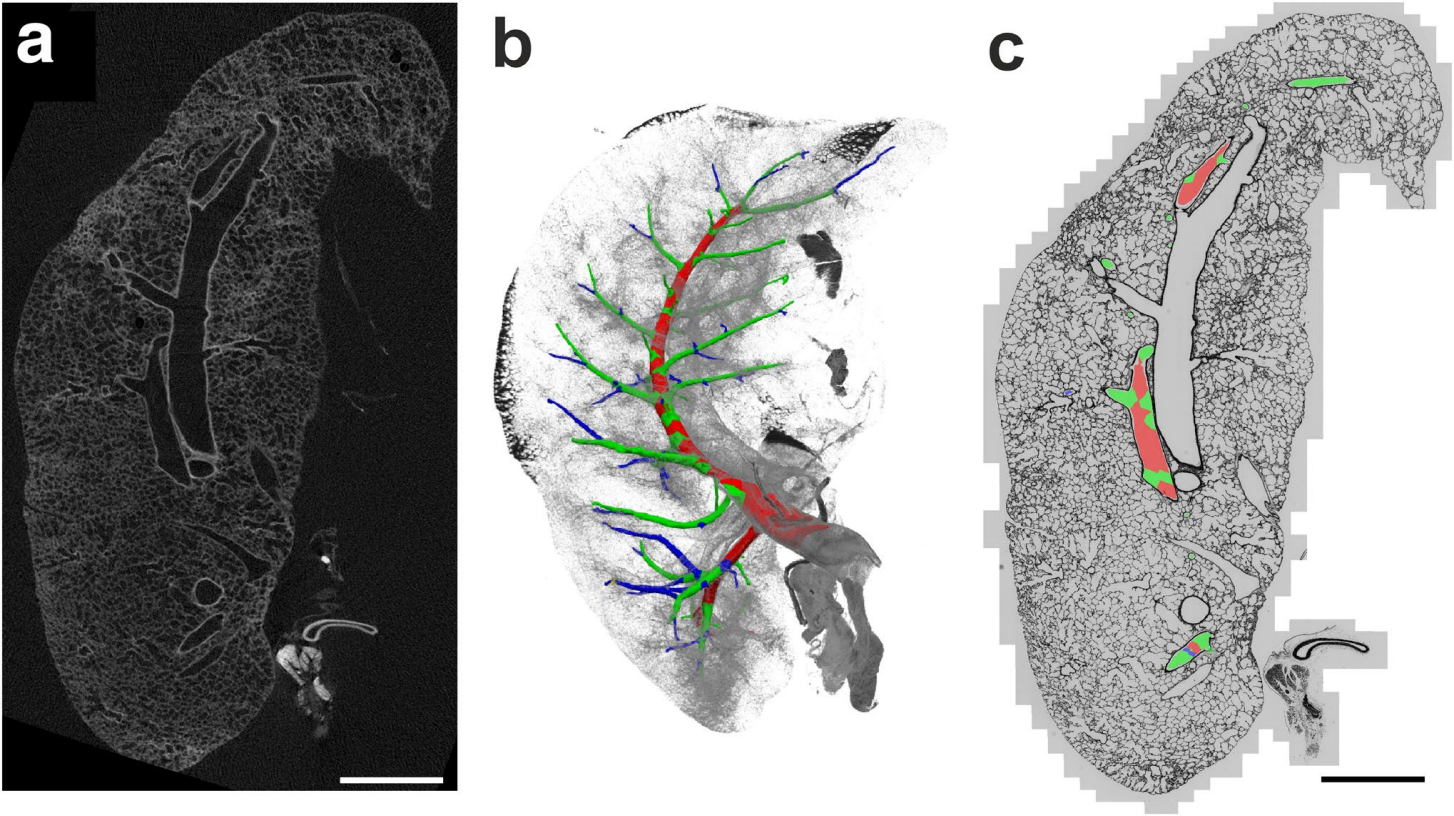
From µCT to light microscopy of the arterial tree. A whole left rabbit lung embedded in glycol methacrylate was imaged by µCT. **a** Slice of the µCT data set. The arterial tree was segmented from the µCT data and visualized in 3D. After segmentation, generations were assigned to the arterial segments and labeled by colors (**b**). The embedded lung was then sectioned exhaustively and sections were registered with the µCT slices as well as the generations of the arterial tree in order to allow analysis of arterial profiles according to their generation within the arterial tree (**c**). Scale bars: 2 mm

With the progression of non-destructive imaging methods and digital segmentation tools, these problems may be overcome. In a recent study from our work group, a new approach was established that is based on the combination of micro-computed X-ray tomography (µCT) and light microscopic stereology (Grothausmann et al. 2021). In short, the left lung of a postnatal rabbit was embedded in a medium that allows later serial sectioning with reproducibly small section thickness (e.g. 2 µm), here glycol methacrylate. Using µCT, a data set of the whole lung was generated to segment the arterial tree and perform a generation analysis assigning a color code to coherent generations. The lung was then sectioned exhaustively and series of 20 consecutive sections were collected every 100 µm and mounted on glass slides. The digitized sections were registered with the µCT data and the results of the generation analysis which makes it possible to perform morphometric analyses on a certain compartment of blood vessels based on their localization within the arterial tree (Fig. 4).

Although the method has been published as a proof of principle, much work is still to be done before it can be used routinely: first of all, the current work process is very time-consuming. The most labor-intensive part is the transfer of the µCT data to the serial light microscopic sections. This step would be unnecessary if the resolution of the µCT was high enough to clearly delineate the arterial walls and allow their morphometric analysis. The potential of the combination of stereology and µCT has been documented already (Vasilescu et al. 2013, 2020) and the use of contrast agents (Hlushchuk et al. 2018; Chadwick et al. 2021) will help to increase the potential of performing stereological analysis of the vascular tree on scans. In addition, other methods of X-ray imaging (such as synchrotron X-ray tomographic microscopy) reach better resolutions and have been used for lung morphometry already (Barré et al. 2014; Schittny 2018; Borisova et al. 2021). However, the microscopic approach still has several advantages that justify the higher workload, in particular the identification of the cellular and extracellular details of the vessel wall as well as the use of certain staining protocols or immunohistochemistry.

The second question that remains to be solved is related to the 3D characteristics of the arterial tree and the definition of an artery belonging to the same vascular compartment. In a strictly dichotomously branching lung every branching point would give rise to a new (theoretically equal) generation of arterial branches. However, when looking at a human lung, such a design would not make sense: it certainly requires a lot more generations from the hilus of the lung until the subpleural parenchyma at the base of the lung than to the perihilar gas-exchange region. According to the dichotomous branching, an average number of 23–28 generations of arteries is widely accepted (Ochs and Weibel 2008). In contrast to the generational approach starting at the central arteries, an alternative classification has been proposed that starts with the peripheral arteries and divides the branches into orders (Strahler 1957; Horsfield 1978). The order classification starts with the vessels of smallest diameter, order 1, and the order increases whenever two vessels of the same order meet. However, when vessels of different order meet, say an order 1 and order 2 artery, the confluent remains as order 2 (Jiang et al. 1994). Both classifications have their merits: the bifurcation approach explains the architecture of the lung in a very systematic way, the order classification is harder to comprehend but explains the arterial tree in a more physiological way. Surprisingly, little research has been devoted to the distinction between the two different concepts, although it is conceivable that they are (patho-)physiologically important, as they lead to different numerical contributions of arterial branches to the pulmonary circulation. The previous considerations are also true for monopodial lungs such as those of rabbits and rodents. In monopodial lungs, a large primary branch traverses the whole lobe and gives rise to a series of secondary branches (Davies and Reid 1991). From the secondary branches, a number of tertiary branches originate and so forth. However, from the described pattern it cannot be concluded that the primary, secondary, etc., branches have the same morphology along their course. Again, the order-based classification starting in the periphery might provide a physiologically more relevant picture than the centrally starting dichotomous approach. Thus, before site-specific stereology can enter a routine workflow, it will be important to perform basic investigations into the branching pattern of the pulmonary arterial tree in various species.

## Concluding remarks

Morphological analyses of the pulmonary vascular trees as well as the alveolar capillary network are essential to improve our functional understanding of the lung vasculature in health and disease. New microscopic and X-ray imaging techniques, digital image processing and stereological estimators increase the potential of microscopic investigations of the pulmonary vasculature. Future studies will benefit from using and combining the techniques to enhance their conclusive potential.

## Data Availability

Not applicable.
